# Editorial: Putting the Spotlight on the Role of Oxygen in Critically-Ill Patients: From Basic Mechanisms to Clinical Effects

**DOI:** 10.3389/fmed.2022.859632

**Published:** 2022-02-25

**Authors:** Stefano Busani, Elena Munari, Fabio Silvio Taccone, Abele Donati, Massimo Girardis

**Affiliations:** ^1^Anesthesia and Intensive Care Unit, University Hospital of Modena Policlinico, University of Modena and Reggio Emilia, Modena, Italy; ^2^Department of Intensive Care, Hôpital Erasme, Université Libre de Bruxelles (ULB), Bruxelles, Belgium; ^3^Anesthesia and Intensive Care Unit, Azienda Ospedaliera Universitaria Ospedali Riuniti, Ancona, Italy; ^4^Department of Biomedical Sciences and Public Health, Università Politecnica delle Marche, Ancona, Italy

**Keywords:** hyperoxia, hyperoxemia, ICU, critically-ill patients, children

Oxygen is vital for life, but its liberal administration sometimes occurs in hospitals without evident pathological prerequisites. Exposure to high oxygen concentrations can potentially enhance the production of reactive oxygen species (ROS), which, if on one hand appear necessary for host defense, on the other hand may result harmful in some acute conditions (1, 2). In recent years, the possible complications related to excessive oxygen administration have been investigated (3); however, in critically ill patients, the critical threshold resulting in subsequent oxygen toxicity remains unclear. The aim of the Research Topic of the articles in this issue, dedicated to critically ill patients, was to outline some interesting issues on this Research Topic, starting from the molecular mechanisms up to different clinical applications. Five articles were submitted to this thematic collection, two of which were basic research, two mini-reviews, and one original research study.

In the preclinical study by Damiani et al. the physiological response to hyperoxia, normoxia, and mild hypoxia was evaluated in spontaneously breathing rats. The authors aimed to evaluate the effects of different inhaled oxygen fractions (FiO_2_) on macrohemodynamics and microvascular perfusion. Results from this experiment showed that the exposure to hyperoxia was characterized by a transient increase in mean arterial pressure (MAP) and systemic vascular resistance (SVR), suggesting ongoing vasoconstriction. On the other hand, mild hypoxia was associated with a reduction in MAP and SVR, with no significant changes in cardiac index. When microcirculation was assessed at the muscular level, the exposure to hypoxia led to a vasodilatory response with an increase in the density of microvascular vessels without modification of the microvascular flow capacity, while microvascular density decreased under hyperoxia.

In contrast, another preclinical study by Li et al. focused on the role of hyperoxia on gut and microbiome injury in mice. In particular, the authors analyzed the time- and the dose-dependent effects of hyperoxia on the gut, using a transcriptome analysis to understand the mechanism of oxygen-induced gut injury related to innate immunity. The mice were randomly assigned to normoxia (control group, room air O_2_) or one of nine hyperoxia groups using different FiO_2_ during different temporal exposure. The results of this study showed that hyperoxia causes time- and dose-dependent gut injury characterized by mucosal atrophy (i.e., villus shortening), enterocyte death, intestinal barrier dysfunction, increase in pro-inflammatory cytokines, and reduction of anti-inflammatory cytokines. The authors proposed the hypothesis of the potential crosstalk between gut dysbiosis, and gut injury induced by hyperoxia. Indeed, inhaled oxygen might promote the expansion of oxygentolerant *Enterobacteriaceae*, which release bacterial lipopolysaccharides (LPS) through impaired tight junctions into the bloodstream, resulting in endotoxemia. LPS from gram-negative bacteria and peptidoglycan from gram-positive bacteria activate innate immunity (Toll-like receptor 4, Nod-like receptor P3, Nucleotide-binding oligomerization domain signaling pathways) leading to gut inflammation and cell death.

The two mini reviews focused on the effects of hyperoxemia in children and hyperoxia in the perioperative setting of adult patients. Pelletier et al. analyzed 12 studies on the association between hyperoxemia and outcomes in critically ill pediatric patients. Six of 12 studies considered heterogeneous patients admitted to pediatric intensive care units (ICUs), highlighting a *U*-shape relationship between oxygen and mortality; among these studies, four showed an increase in mortality with PaO_2_ > 300–400 mmHg, and only one >550 mmHg. Four other studies focused on hyperoxemia in pediatric ICU patients after cardiac arrest; of these, only one demonstrated that hyperoxemia was associated with increased mortality, while the other three did not. A single retrospective study included ICU patients with severe traumatic brain injury, and it showed that PaO_2_ admission of 300–500 mmHg was associated with increased survival at discharge. However, this study enrolled patients with concomitant chest trauma, which may be a confounding factor on the beneficial effect of increased PaO_2_ in traumatic brain injury. The optimal cut-off at which hyperoxemia was significantly increase the risk of harmful effects remained unclear; according to these studies, this cut-off ranged between 250 and 400 mmHg, demonstrating as well that it may subsist a polynomial “U-curve” relationship between PaO_2_ and mortality in pediatric critically ill patients, with both hypoxemia and hyperoxemia associated with increased mortality. Busani et al. described the pathophysiological effects of hyperoxia on different organs in the surgical setting. The Research Topic is somewhat controversial because the World Health Organization guidelines still recommend the administration of 80% FiO_2_ to reduce surgical site infections in adult patients undergoing general anesthesia; on the other hand, growing pathophysiological evidence showed that hyperoxia causes deleterious effects on many organs. An elevated systemic oxygen supply could induce oxidative stress with inflammation, vasoconstriction, impaired microcirculation, activation of haemostasis, acute and chronic lung injury, coronary blood flow disturbances, cerebral ischemia, gut dysbiosis, and altered antibiotics susceptibility. Regarding the prevention of surgical wound infections, it has been demonstrated that ROS alter the actin cytoskeleton of macrophages and their antibacterial function. For all these reasons, the authors suggested that the maintenance of PaO_2_ within physiological limits during the perioperative process might be the best option.

Finally, Justus et al. conducted an exploratory, single-center, retrospective cohort study on critically ill patients on extracorporeal veno-arterial membrane oxygenation (VA-ECMO). The authors focused on the association between oxygenation, removal of carbon dioxide (PaCO_2_) and mechanical ventilation setting with in-hospital mortality. Fifty-two patients were included in the analysis and 50% were alive at the time of hospital discharge. Compared with non-survivors, hospital survivors had a significantly lower ECMO blood flow rate and received significantly higher native lung minute ventilation. Hospital survivors had significantly lower cumulative mean PaO_2_ than non-survivors (117 vs. 154 mmHg, *p* = 0.04). Hospital survivors also had significantly higher cumulative mean PaCO_2_ than hospital non-survivors. However, on multivariable logistic regression analysis, hyperoxemia, PaCO_2_, and minute ventilation were not independently associated with in-hospital mortality. The authors highlighted that hyperoxemia was common in this cohort mainly in the first 48 h of treatment, however their data did not show increased mortality in contrast to other reports that showed independent higher mortality. Rather, they concluded that interactions between hyperoxemia, PaCO_2_, and minute ventilation was more relevant than their individual association with survival.

Hence, in conclusion, the data and reviews published in this Research Topic have shown that hyperoxia is burdened by a series of deleterious effects in critically ill patient. However, it is not possible to make any kind of recommendation as the evidence are not yet conclusive on this debated Research Topic.

## Author Contributions

SB, EM, and FT revised the articles belonging to the Research Topics and wrote the original version of the Editorial. AD and MG revised the Editorial. All authors have contributed to the manuscript. All authors have read and approved the final version of the manuscript.

## Conflict of Interest

The authors declare that the research was conducted in the absence of any commercial or financial relationships that could be construed as a potential conflict ofinterest.

## Publisher's Note

All claims expressed in this article are solely those of the authors and do not necessarily represent those of their affiliated organizations, or those of the publisher, the editors and the reviewers. Any product that may be evaluated in this article, or claim that may be made by its manufacturer, is not guaranteed or endorsed by the publisher.
